# …And justice for all: justice sensitivity outweighs empathy in pro-environmental behavior

**DOI:** 10.3389/fpsyg.2026.1712314

**Published:** 2026-03-23

**Authors:** Susanne Nicolai, Anette Hiemisch, Susanne Kleemann

**Affiliations:** 1 Institute of Geography and Geology, University of Greifswald, Greifswald, Germany; 2 Institute of Psychology, University of Greifswald, Greifswald, Germany

**Keywords:** pro-environmental behavior, justice sensitivity, cognitive empathy, affective empathy, compassion

## Abstract

Extensive psychological research has documented that becoming aware of injustice, coupled with a negative evaluation of it, motivates actions aimed at addressing the injustice. Perceiving environmental harm as unjust—because it disproportionately affects those least responsible—may therefore motivate pro-environmental behavior (PEB). This study investigates whether empathy contributes to this process via perspective taking and empathic concern. We investigated the interrelationships of justice sensitivity (JS) dimensions (victim, observer, beneficiary, and perpetrator sensitivity) with three facets of empathy (cognitive empathy, affective empathy, and compassion) and PEB. A preregistered study (*N* = 327) showed that other-oriented forms of JS were positively associated with PEB, while empathy facets showed no significant relationships. No interaction effect was found. These findings suggest that making justice concerns more salient may increase PEB. Future research should further investigate the psychological mechanisms through which JS supports PEB and how it can be activated in communication and intervention strategies. The role of affective responses in this process is examined.

## 1 Introduction

The phrase “And justice for all” is widely recognized as a powerful appeal to fairness and equality—popularized both by the American Pledge of Allegiance and, more recently, as the title of a song and album by the heavy metal band Metallica. It captures a central moral principle: justice that extends beyond the self. In the face of climate injustice, this principle gains new relevance.

Activists, researchers and politicians around the world are calling for climate justice. A term that describes the need for distributive justice (global fair sharing of costs and benefits), procedural justice (opportunities to participate, for example, in law-making processes) and interactional justice (respectful communication and recognition of all cultures, ages, genders, religions, etc.) in the face of ongoing climate change (Calvin et al., 2023; IPCC, 2022). But how can climate justice be achieved? Psychological research suggests that the perception of injustice is a strong motivator for both moral behavior (Baumert et al., 2013) and pro-environmental behavior (van Zomeren et al., 2008). Therefore, the goal might be the way to achieve it at the same time. This study examines whether individual differences in other-oriented justice sensitivity and compassion are associated with pro-environmental behavior, and whether compassion moderates the relationship between justice sensitivity and such behavior. Understanding these associations may provide insights into the moral motivational basis of environmental action and inform climate communication strategies.

### 1.1 Facilitators of pro-environmental behavior

Since most strategies for mitigating (and adapting to) climate change require behavior change (IPCC, 2022), promoting pro-environmental behavior is a key component of climate action. By adopting sustainable lifestyles—such as reducing consumption and supporting clean energy—individuals and societies can significantly lower their ecological footprint. Such individual and collective efforts not only contribute to the mitigation of climate change but also advance climate justice, as they uphold the rights of vulnerable populations to a safe and stable environment. In this way, pro-environmental behavior becomes a crucial lever for addressing the root causes of environmental degradation and for reducing the disproportionate impacts of climate change.

Pro-environmental behavior includes all those behaviors of an individual that have a positive effect on our environment (Steg and Vlek, 2009; Stern, 2000). In addition to civic engagement or financial investments, relevant areas include nutrition, mobility, waste management, consumption of resources such as energy and water, and purchasing behavior (Schultz and Kaiser, 2012). Pro-environmental behavior is most effective, when it is (1) widespread—many people do it (Stern, 2000), (2) socially influential—an individual acts as a role model and inspires others around them to behave in a similar way (according to social learning theory, Bandura and Walters, 1963), or (3) strategically targeted—behaviors with a great leverage are changed (Nielsen et al., 2021; Stern, 2000). For instance, energy use in private households and voting behavior are two areas where individual actions can have substantial impact. In 2018, household energy consumption alone accounted for a quarter of total EU energy consumption (Tsemekidi Tzeiranak et al., 2020). Furthermore, a study in Germany shows that the likelihood of an environmental action plan being drawn up increases with the strength of the Green Party in the local council (Schulze and Schoenefeld, 2022). However, understanding what drives people to engage in such behaviors is complex and cannot be explained by single factors alone.

Whether or not someone behaves pro-environmentally and to what degree, depends on a variety of (sometimes interacting) factors (e.g., Bamberg, 2013; Rau et al., 2022). For this reason, it is important not only to identify individual determinants but also to explore how they are interconnected (Gifford and Nilsson, 2014; Stern, 2000; Stoll-Kleemann and Nicolai, 2024). Adopting such an integrative perspective enables a more comprehensive understanding of the mechanisms that shape behavior. In line with this, the present study examines both the direct associations of justice sensitivity and empathy with pro-environmental behavior, as well as their potential interactions and underlying dynamics.

### 1.2 Perception of injustice

As justice is a fundamental human motive (Baumert et al., 2013; Montada, 2007), people are generally motivated to support justice and prevent injustice (Lerner, 1975). This motivation reflects a concern for fair treatment of both oneself and others and includes a tendency to behave in ways that are consistent with justice-related norms and values (Baumert, 2013). To achieve justice, individuals are willing to sacrifice their own benefits (Engel, 2011), engage in sustainable behavior (Kals and Becker, 1994), or reject a favorable but unfair deal (Sutter et al., 2020). Strikingly, perceived injustice is not only a predictor of pro-environmental attitudes (Kals, 1996), but also a significant driver of political engagement and collective action, as a meta-analysis of 182 studies shows (van Zomeren et al., 2008). Moreover, it was found crucial in resolving environmental conflicts (Lofstedt, 1996).

People differ in how and to which extend they perceive injustice. The concept of justice sensitivity describes a dispositional tendency to perceive and respond more or less strongly to injustice (Baumert and Schmitt, 2009). In individuals with higher justice sensitivity, ambiguous information is more frequently interpreted as unjust, and the memory for injustice-relevant information is better than that of less justice sensitive individuals. Additionally, individuals who score high on justice sensitivity exhibit increased selective attention to unjust situations (Baumert et al., 2011; Schmitt et al., 2009). Injustice sensitivity is a stable trait whose predictive value goes beyond that of established personality dimensions, such as those in the HEXACO model (Stavrova and Schlösser, 2015).

Injustice can be perceived either as directed at oneself (victim sensitivity) or at others (perpetrator, beneficiary, and observer sensitivity; Baumert et al., 2011; Schmitt et al., 2010). While the four perspectives are conceptually distinct (Schmitt et al., 2010), they are moderately interrelated due to a shared underlying concern for justice (Bondü et al., 2022). Although individuals can adopt different perspectives depending on the context, they typically show a stronger sensitivity to one. Each perspective is linked to specific emotional reactions and behavioral responses aimed at restoring justice (Schmitt et al., 2009). Among the four perspectives, beneficiary and perpetrator sensitivity share the most conceptual similarities. Both are linked to feelings of guilt (Montada et al., 1986). Individuals high in beneficiary or perpetrator sensitivity often show compassionate reactions toward disadvantaged groups (Gollwitzer et al., 2005). Like beneficiary, observer sensitivity is positively associated with socially desirable traits such as fairness (Fetchenhauer and Huang, 2004) and solidarity (Gollwitzer et al., 2005). Yet, observer sensitivity is typically accompanied by moral outrage (Schmitt et al., 2009). Victim sensitivity describes individual differences in emotional reactivity—especially anger—to perceived personal disadvantage, even when the injustice is not objectively evident (Schmitt et al., 2009; Süssenbach and Gollwitzer, 2015). It reflects a stable response tendency and is linked to negative social and cognitive patterns such as suspicion, social withdrawal, hostile attribution, prejudice, envy, and the expectation of future injustice (Bondü et al., 2022; Gerlach et al., 2012; Hong et al., 2023).

The perspectives of injustice sensitivity differ from each other in terms of prosocial, altruistic, and cooperative behavior. While observer, perpetrator, and beneficiary sensitivity have been repeatedly associated with cooperative behavior, the opposite is true for victim sensitivity (e.g., Stavrova and Schlösser, 2015). Overall, victim sensitivity primarily reflects a self-focused concern with justice, often characterized as egoistic (Gollwitzer and Rothmund, 2011). In contrast, the other three perspectives—perpetrator, beneficiary, and observer sensitivity—are considered expressions of a broader, more genuine concern for justice as a moral principle (Gollwitzer et al., 2009; Gollwitzer and Rothmund, 2011). Therefore, the three other-oriented sensitivities are sometimes combined into a factor of “sensitivity for others” (Fetchenhauer and Huang, 2004).

Perceiving an injustice can elicit different types of affective reactions. In addition to direct emotional reactions—such as anger or guilt—it may also evoke feelings of empathy or concern for the person affected. On the one hand, the degree of empathy with the victim might amplify the impact of justice sensitivity: the more strongly individuals emotionally identify with the victim, the more intense their response to the injustice may tend to be. On the other hand, individuals with heightened sensitivity to injustice may be more inclined to adopt the perspective and perceive the emotions of victims and feel a stronger motivation to address or rectify their situation.

### 1.3 Empathy

The concept of empathy remains somewhat vage, as its definitions vary across the psychological literature (Decety and Cowell, 2014). In line with the recommendations by Cuff et al. (2016), we define empathy as the capacity to recognize and share the emotional states of others, whether directly perceived or inferred. Crucially, this process requires a clear distinction between one’s own emotional experience and that of the other person (Brett et al., 2023; Singer and Lamm, 2009). Empathy is commonly conceptualized as a multidimensional construct comprising cognitive, affective, and motivational components (Brett et al., 2023; Davis, 1983; Decety and Cowell, 2014), each of which contributes differently to social behavior (Bloom, 2017).

Cognitive empathy refers to the ability to understand another person’s thoughts, beliefs, and emotions without necessarily sharing their emotional experience; it is sometimes equated with theory of mind (e.g., Leslie et al., 2004). In contrast, affective empathy involves sharing or mirroring the emotional state of others (Bloom, 2017). Closely related but conceptually distinct is compassion—or empathic concern—which refers to other-oriented emotional response characterized by feelings of warmth, care for others’ wellbeing and typically involves a motivation to alleviate their suffering (Goetz et al., 2010; Pfattheicher et al., 2016).

Compassion is fundamentally defined as an emotional response to the suffering of others, aiming to alleviate their distress and enhance their wellbeing (Pfattheicher et al., 2016). It reflects a broader moral orientation toward preventing harm and promoting the welfare of others, and is therefore closely tied to a concern for humanity (Goetz et al., 2010; Sprecher and Fehr, 2005). Empirical findings further support this view by linking compassion to prosocial behaviors such as helping vulnerable individuals, volunteering, and contributing to collective causes (Batson et al., 1983; Eisenberg, 1988, 2010). Given this orientation toward the needs of others, it appears plausible that compassion is positively associated with other-oriented justice sensitivities which also reflect a heightened awareness of and responsiveness to injustices experienced by others.

Several theoretical models address the relationship among these components: Some propose that affective empathy is a necessary precursor to compassion, which then motivates prosocial behavior (Singer and Lamm, 2009). Supporting this, empirical studies suggest that both affective and cognitive empathy contribute to the development of complex social cognitions like compassion and respect (Lim and DeSteno, 2016; Malti et al., 2020). In contrast, other perspectives argue that compassion primarily follows from cognitive empathy—specifically, the capacity to adopt another’s perspective (Eisenberg, 2010). Despite these theoretical differences, there is broad agreement that compassion represents the most advanced empathy facet and is closely linked to cognitive empathy. However, the precise nature of the relationship between affective empathy and compassion remains unclear (de Waal, 2008; Malti et al., 2020; Singer and Lamm, 2009). While these models are not directly tested here, they provide important context for understanding the complexity of empathy facets in relation to justice sensitivity and behavior.

Importantly, several authors emphasize that empathic concern—the affective component of empathy characterized by feelings of warmth, compassion, and care for others—plays a particularly central role in motivating prosocial behavior. For example, Decety and Cowell (2014) argue that empathic concern facilitates emotion-driven decision-making processes that promote helping behavior. In line with this view, affective responses to others’ suffering may be especially relevant for actual behavioral engagement, as they generate an immediate motivational impulse to reduce perceived harm (Batson et al., 1983). Applied to the environmental domain, such affectively grounded concern for others (= compassion toward affected individuals) affected by climate change may therefore translate not only into pro-environmental intentions but into concrete pro-environmental actions.

In the context of climate change, this distinction may be especially important. The consequences of climate change disproportionately affect vulnerable populations, linking environmental degradation to issues of social injustice. When climate change is framed as a matter of global justice, concern for the wellbeing of others—particularly those in the Global South or future generations—may therefore serve as a motivational basis for pro-environmental behavior.

In research on sustainability and pro-environmental behavior, the concept of empathy often differs from that adopted here. Many studies focus on empathy toward non-human entities, such as plants or animals (e.g., Berenguer, 2007; Tam, 2013). This raises conceptual challenges: emotions elicited by, for example, observing a damaged tree do not imply that the tree experiences emotions or thoughts akin to human observers, and thus these responses are not empathy per se. Furthermore, these feelings may arise from recognizing environmental harm’s personal consequences or from a deep connectedness to nature. Grouping these diverse responses under “empathy” risks conceptual confusion and complicates understanding their distinct effects on behavior. To avoid conceptual ambiguity, the present study focuses specifically on empathy toward other humans.

Building on this framework, the present study primarily examines whether compassion—as an other-oriented affective response characterized by concern for others’ suffering—is associated with other-oriented justice sensitivity and pro-environmental behavior, and whether compassion moderates the relationship between justice sensitivity and behavioral engagement. Given its explicit motivational component, compassion is expected to play a particularly central role in translating justice-related perceptions into action.

To provide a more comprehensive understanding of empathy’s multidimensional structure, the study additionally considers cognitive and affective empathy. Examining these facets alongside compassion allows for a differentiated analysis of how distinct empathy components relate to justice sensitivity and pro-environmental behavior, thereby clarifying whether action-relevant effects are specific to compassion or extend to other empathy dimensions. By doing so, the study aims to refine the understanding of affective and cognitive moral processes in shaping environmental action.

### 1.4 Current literature

To date there are only a few studies, that address the links between justice sensitivity and pro-environmental behavior. Moreover, some of them found contradictory evidence. Köhler et al. (2024) found positive correlations between perpetrator, beneficiary, and observer sensitivity with pro-environmental attitude, behavior, protest and policy support. The associations between the pro-environmental outcomes and victim sensitivity were not significant in a full mode, that included all justice sensitivity predictors; however, in separate multiple regression analyses including only victim sensitivity, it was consistently found to have a negative correlation with pro-environmental behavior, protest and policy support. Nicolai et al. (2022) found that observer sensitivity significantly predicted pro-environmental intention but not pro-environmental behavior in one study, whereas in a second study, perpetrator sensitivity positively and beneficiary negatively predicted pro-environmental intention in multiple regression analyses. The effects of all justice sensitivity perspectives varied depending on the inclusion of other predictors (e.g., moral disengagement) into the regression model. Although the four justice sensitivity perspectives are theoretically distinct, their substantial common ground may obscure their individual effects in regression analyses. Consequently, this study aims to examine each perspective separately to better account for the conceptual richness and multidimensionality of justice sensitivity. To this end, we investigate zero-order correlations between the four justice sensitivity facets and pro-environmental behavior.

Compassion was found to be associated with support for government action to mitigate climate change (Lu and Schuldt, 2016), and more pro-environmental decision making (Engel et al., 2021; Zelenski and Desrochers, 2021). Dispositional compassion has been associated with and induced compassion has led to greater intention to engage in pro-environmental behaviors (e.g., recycling, water and energy conservation, green purchasing, and green transportation) (Pfattheicher et al., 2016). Due to the other-orientation inherent in the definition of compassion (Pfattheicher et al., 2016) and its links to prosocial behaviors as well as community-orientation (Batson et al., 1983; Eisenberg, 1988, 2010), we hypothesize that compassion is positively related to the justice sensitivities for others.

A recent study by Ienna et al. (2022) found that cognitive empathy significantly and positively predicted preservation behavior. In contrast, affective empathy showed a non-significant, slightly negative association with preservation behavior. These findings suggest that similar patterns may also apply to pro-environmental behavior more broadly. Breithaupt’s (2019) concept of the “dark side” of empathy may also help explain this finding: When individuals emotionally align with one side of a conflict or moral scenario, they may justify harmful inaction or maintain a distorted moral self-image. Such mechanisms could blunt the potential of empathy to motivate genuine prosocial—or pro-environmental—action, particularly when affective empathy dominates without reflective distancing.

Given that compassion is closely linked to costly *pro-social* behavior (FeldmanHall et al., 2015), it can be assumed to involve a particularly strong motivational component for other forms of behavior as well. Accordingly, we hypothesize that compassion will emerge as the strongest predictor of *pro-environmental* behavior among the various empathy facets. Cognitive empathy is also expected to positively relate to pro-environmental behavior, as it has been shown to foster both prosocial and pro-environmental tendencies (Ienna et al., 2022; Yin and Wang, 2023), although presumably to a lesser extent than compassion. By contrast, we expect affective empathy to show little or no association with pro-environmental behavior. Previous research has found its relation to prosocial outcomes to be inconsistent, with some evidence suggesting that heightened affective empathy may even diminish prosocial engagement (Stevens and Taber, 2021). Moreover, affective empathy has been identified as a positive predictor of victim sensitivity (Decety and Yoder, 2016), which is itself negatively associated with pro-environmental behavior.

Empirical research further highlights complex associations between empathy and justice sensitivity. Studies indicate positive correlations between empathy and PS, BS, and OS (Bondü et al., 2022). When these three sensitivities are combined into a composite construct—“sensitivity for others”—this is positively predicted by perspective taking (“cognitive component”) and empathic concern (“motivational component”), but not significantly by personal distress (“affective component”) (Decety and Yoder, 2016). Conversely, “sensitivity for the self” (VS) is negatively predicted by empathic concern, positively predicted by personal distress, and unrelated to perspective taking. These findings suggest that affective empathy aligns with the self-centered victim perspective, whereas cognitive empathy and compassion are linked to other-oriented justice sensitivities. However, different from the conceptualization of Decety and Yoder (2016) the superdimension of affective empathy under Davis (1983) IRI encompasses both empathic concern and personal distress subscales, not only personal distress. Therefore, operationalization differences in empathy measures may influence these results. Hence, the current study aims to examine the nuanced relationships between justice sensitivity facets and empathy dimensions more closely.

Many authors combine the three other-oriented justice sensitivities (perpetrator, beneficiary, observer) into a factor called “sensitivity for others” (e.g., Decety and Yoder, 2016), as they often show similar associations. We will first take a look at individual correlations between the different justice sensitivities and pro-environmental behavior as well as empathy. Based on the results, we then decide if and which of the other-oriented justice sensitivities we combine for the following hypotheses.

Given the relevance of other-oriented justice sensitivity as a predictor of pro-environmental behavior, the question arises as to whether this sensitivity can be systematically influenced. While other-oriented justice sensitivity reflects sensitivity to moral norm violations, compassion involves a motivational component aimed at alleviating suffering. Compassion may therefore strengthen the association between other-oriented justice sensitivity and pro-environmental behavior by transforming moral concern into prosocial action. Moreover, we assume that adopting another’s perspective—a core element of cognitive empathy—may serve as a mechanism through which other-oriented justice sensitivity can be oriented toward pro-environmental goals. From this perspective, fostering empathy may represent a promising approach for further pro-environmental interventions. Evidence from intervention studies, particularly in medical and caregiving contexts, shows that empathy can be enhanced through targeted training (van Teding Berkhout and Malouff, 2016). Building on this, we propose that empathy training may ultimately increase pro-environmental behavior, by amplifying the effect of justice sensitivity. To derive more specific recommendations for intervention programs—particularly regarding which facets of empathy should be targeted—we additionally examine potential interaction effects involving affective empathy. Given that personal distress is primarily associated with victim sensitivity (Decety and Yoder, 2016), we exploratorily hypothesize that affective empathy amplifies the negative association between victim sensitivity and pro-environmental behavior. In our preregistration, the moderation hypotheses were formulated in the opposite direction, reflecting our initial focus on empathy training. However, as the focus of this article has shifted toward justice sensitivity, it now appears more informative to examine whether empathic ability amplifies the effect of justice sensitivity. As this deviates from the preregistration, the three moderation hypotheses are treated as exploratory.

### 1.5 Hypotheses

Taking together, we assume that pro-environmental behavior is primarily driven by other-oriented moral motivation rather than self-oriented sensitivity. We focus on justice sensitivity and compassion as core moral orientations and examine cognitive and affective empathy as additional psychological mechanisms. Our main hypotheses are:

*H1*: Other-oriented justice sensitivities (perpetrator, observer, beneficiary) are positively associated and victim sensitivity is negatively associated to pro-environmental behavior.

*H2*: Compassion is positively associated with pro-environmental behavior.

*H3*: The association between other-oriented justice sensitivity and pro-environmental behavior is moderated by compassion.

Additionally, we had the following secondary hypotheses:

*H4*: Cognitive empathy is positively associated with other-oriented justice sensitivities and negatively with victim sensitivity.

*H5*: Affective empathy is negatively associated with other-oriented justice sensitivities and positively with victim sensitivity.

*H6*: Cognitive empathy is positively and affective empathy is negatively associated with pro-environmental behavior.

*H7*: Compassion is positively associated with other-oriented justice sensitivities and negatively with victim sensitivity.

*H8*: The highest association with pro-environmental behavior shows compassion, followed by cognitive empathy and then affective empathy (last one also negatively).

*H9*: The association between other-oriented justice sensitivity and pro-environmental behavior is moderated by cognitive empathy.

*H10*: The association between victim sensitivity and pro-environmental behavior is moderated by affective empathy.

## 2 Methods

This study was preregistered. Preregistration can be found at: https://aspredicted.org/95xg4.pdf.

### 2.1 Sample

Participants were recruited from various lectures across all faculties as well as via flyers in the university canteen at the University of Greifswald. Participation was voluntary, with the opportunity to win one of ten book vouchers through a raffle. Power analyses were conducted for all calculations and can be found in the preregistration. The highest necessary sample size was *N* = 330, according to the preregistered stopping rule, data collection was terminated when 559 participants had used the link. This higher sample size was to compensate for possible exclusions. Due to the preregistered exclusion criteria, we excluded 232 participants, because they were too young, did not answer the three control items correctly, or their completion time was not within a reasonable range (see preregistration). Thus, 327 participants between 18 and 69 years (*M*_Age_ = 27.2 SD_Age_ = 10.5) were included in the analysis. Of these, 231 identified as female, 79 as male, 7 as diverse, and 10 preferred not to specify any gender. In addition, 238 participants reported being in education or currently enrolled as students. We did not include questions on ethnicity.

Participants were informed about the study’s objectives and data processing procedures as well as the option to opt-out at any time of the survey in advance, without any consequences. Each participant provided explicit consent to the study’s terms and agreed to the utilization and storage of their anonymized data. The given study aim was to explore individuals’ evaluations of specific behaviors and emotions. The concrete information regarding asking about pro-environmental behavior and empathy was debriefed at the conclusion of the study, and participants were offered the option to receive the study’s outcomes. Contact information for the book voucher lottery and interest in receiving study results were gathered separately.

### 2.2 Instruments

#### 2.2.1 Pro-environmental behavior

The General Environmental Behavior (GEB) scale (Kaiser, 2020) is a recognized measurement tool for assessing a person’s tendency to engage in environmentally friendly behavior across various areas (Lange and Dewitte, 2019). The test consists of two parts with a total of 50 items from the behavioral areas (1) energy saving, (2) mobility, (3) waste avoidance, (4) consumption, (5) recycling and (6) social engagement. The first part contains 32 statements about the frequency (“never,” “rarely,” “occasionally,” “often,” and “very often”) of environmentally protective behaviors and the remaining 18 items require a yes/no statement. If a behavior does not apply to the respondent’s life situation, e.g., in the case of the question about driving behavior on freeways for people who do not have a driver’s license, the answer can be “no answer“. An example item for this questionnaire is “I boycott products from companies that are proven to be harmful to the environment.” 19 of the 50 behavioral self-reports are worded negatively. The resulting scale is calculated using a Rasch model. Person separation reliability^1^ was 0.69 and is therefore comparable to the typical reliabilities between rel = 0.71 and rel = 0.88 (Scheuthle et al., 2005).

#### 2.2.2 Justice sensitivity

The USS-8 (Beierlein et al., 2012) for assessing the four perspectives of justice sensitivity comprises a total of 8 items, i.e., two items per perspective. The items were answered on a six-point rating scale from “strongly disagree” (1) to “strongly agree” (6). For each perspective, the mean of two items is calculated, so that a total of four scale values are formed (victim, observer, perpetrator, and beneficiary sensitivity). An example of observer sensitivity is: “It bothers me when someone has to work hard for things that come easily to others”, while an example of victim sensitivity is: “It annoys me when others are undeservedly better off than me”. Cronbachs *α* was acceptable and good except for observer sensitivity (perpetrator: 0.78, beneficiary: 0.87, observer: 0.69, victim: 0.73).

#### 2.2.3 Cognitive and affective empathy

The E-Scale (Leibetseder et al., 2007) is an instrument to measure the trait components of both, cognitive and affective empathy (elicited by real or virtual situations). In this study, we only used the items of real situations. 12 items, with six items for each scale were answered on a 5-point rating scale from “does not apply at all” (1) to “strongly applies” (5) and the mean was used to calculate the two scales. An example item for cognitive empathy is the following “When I see a very old person, I often wonder how I would feel in his/her place”. One for affective empathy is this “It makes me sad to see a lonely person in a group”. Cronbachs α was acceptable for affective (0.71) and questionable for cognitive empathy (0.63).

#### 2.2.4 Cognitive empathy task

We included the German 10-Items-Version of the Mind-In-The-Eye-Test (MITE, Olderbak et al., 2015) as an additional (and non-self-reported) operationalization, but were skeptical regarding what it actually measures (Theory of Mind or cognitive empathy), as well as its internal consistencies. The MITE was invented to measure the ability to attribute mental states to others based on photographs of the eye region of different actors and actresses. Correct answers are summed together to calculate a score for each participant. As internal consistency (*α* = 0.26) was very low, we did not use it for further analyses.

#### 2.2.5 Compassion

Compassion was measured in this study by the COS-7 (Schlosser et al., 2023), a short scale of compassion for others (strangers) comprised of items from the Compassionate Love Scale (CLS; Sprecher and Fehr, 2005). It consists of seven items, that uses a 7-point rating scale ranging from 1 (“not at all true of me”) to 7 (“very true of me”). An example item for this scale is: “If I met a stranger who needed help, I would do almost anything I could to help them”. The total score is derived by averaging all item scores. Standardized Cronbachs´ α was good (0.86).

#### 2.2.6 Moral orientation scale

Additionally, the Moral Orientation Scale (MOS, Hiemisch et al., 2026) was surveyed, which is not included in the hypotheses and analyses of this study.

#### 2.2.7 Attention checks

Three questions were hidden in between the survey, which asked “if you read this please click [yes (GEB), neither (E-Scale), one (MOS)]”).

Sociodemographic variables such as gender, age, and working situation were queried directly.

### 2.3 Design and procedure

We conducted the study online via Sosci-Survey (Leiner, 2021). After providing their explicit consent, the participants answered the questionnaires in the following order: GEB, MOS^2^, USS-8, MITE, COS-7, E-Scale, Sociodemographic variables. Answering all questions took the participants between 10 and 42 min (Mean = 19 min).

The findings were considered significant when *p* < 0.05. Datascreening, calculation of scales, and all analyses were conducted using R (R Core Team, 2022). In addition to established packages, the following were used: dplyr (Wickham et al., 2022), and eRM (Mair et al., 2021). For an *a priori* sample size calculation and calculation of effect sizes, we used G*Power (Faul et al., 2007).

The data set is available at: https://osf.io/j5q7h/files/osfstorage.

### 2.4 Ethics statement

Data from human participants were collected in this study. No formal ethics committee approval was needed, as the study involved no intervention or experimental treatment and consisted solely of non-invasive, self-report questionnaires. All participants received comprehensive information about the study and data processing in accordance with the EU General Data Protection Regulation (GDPR) and provided explicit informed consent prior to participation, confirming that they were of legal age, voluntarily agreed to participate, and consented to the storage and use of their data for research purposes. Participants were able to stop, pause, and leave the questionnaire at any time.

## 3 Results

The descriptive statistics of all variables are depicted in Table 1.

**Table 1:** Descriptive statistics of all variables.

Variable	Min	Max	*M*	SD
Pro-environmental behavior	−2.47	3.11	0.63	0.92
Perpetrator sensitivity	1	6	4.93	1.13
Beneficiary sensitivity	1	6	3.69	1.29
Observer sensitivity	1	6	4.29	1.06
Victim sensitivity	1	6	3.59	1.15
Cognitive empathy	1	5	3.27	0.74
Affective empathy	1	5	3.64	0.53
Compassion	1.29	7	4.68	1.05
Cognitive empathy test (MITE)	2	10	7.57	1.50

*N* = 327.

To investigate hypotheses 1, 2, and 4–7, we calculated zero-order correlations. As Shapiro–Wilk-Tests showed normal distribution only for pro-environmental behavior, both, pearson and spearman rank correlations were applied. As shown in Table 2, the results supported the assumed relationship of justice sensitivity and pro-environmental behavior: the three other-oriented justice sensitivities—perpetrator, beneficiary, and observer sensitivity—were positively associated with pro-environmental behavior, with perpetrator sensitivity emerging as the strongest predictor. In contrast, self-oriented victim sensitivity was negatively associated with pro-environmental behavior (Hypothesis 1). Therefore, our first main hypothesis was supported. However, our findings could not support the other two main hypotheses on compassion, as it was not related with pro-environmental behavior and did not moderate the relationship between other-oriented justice sensitivity and pro-environmental behavior, as the multiple regression model 1 in Table 3 shows. Nevertheless, compassion was positively associated with other-oriented justice sensitivity, though victim sensitivity did not show a notable negative correlation with it (Hypothesis 7).

**Table 2:** Spearman rank correlations to test for hypotheses 1–2 and 4–7.

Variable	PS	BS	OS	VS	CE	AE	CM
Pro-environmental behavior	0.32^***^	0.24^***^	0.22^***^	−0.11^!^	−0.08	0.11^!^	0.09
Perpetrator sensitivity (PS)		0.51	0.38	0.01	0.17^**^	0.25^***^	0.25^***^
Beneficiary sensitivity (BS)			0.50	0.19	0.15^**^	0.20^***^	0.26^***^
Observer sensitivity (OS)				0.35	0.22^***^	0.30^***^	0.29^***^
Victim sensitivity (VS)					0.20^***^	0.09	−0.01
Cognitive empathy (CE)						0.46	0.45
Affective empathy (AE)							0.41

CM, Compassion. *N* = 327. ^*^p < 0.05, ^**^p < 0.01, ^***^p < 0.001, ^!^*p* = 0.05.

**Table 3:** Regression analyses to calculate hypothesized moderation effects (exploratory hypotheses 3, 9, and 10).

Coefficient	*β*	95% CI	*p*
Model 1 (Hypothesis 3)			
Intercept	−0.70	[−2.22, 0.82]	0.37
Compassion	0.02	[−0.32, 0.36]	0.92
Other-oriented sensitivity	0.29	[−0.08, 0.66]	0.13
Compassion * Other-oriented sensitivity	0.00	[−0.08, 0.08]	0.99
Model 2 (Hypothesis 9)			
Intercept	0.38	[−1.27, 2.04]	0.65
Cognitive empathy	−0.38	[−0.91, 0.15]	0.16
Other-oriented sensitivity	0.21	[−0.18, 0.60]	0.29
Cognitive empathy * Other-oriented sensitivity	0.04	[−0.08, 0.16]	0.51
Model 3 (Hypothesis 10)			
Intercept	−1.15	[−3.40, 1.10]	0.31
Affective empathy	0.59	[−0.03, 1.22]	0.06
Victim sensitivity	0.25	[−0.34, 0.84]	0.41
Affective empathy * Victim sensitivity	−0.01	[−0.26, 0.07]	0.24

Model 1: *F*(3, 323) = 11.07, *p* < 0.001, adj. *R*^2^ = 0.08; Model 2: *F*(3, 323) = 14.75, *p* < 0.001, adj. *R*^2^ = 0.11; Model 3: *F*(3, 323) = 4.04, *p* < 0.01, adj. *R*^2^ = 0.03.

Some findings were contrary to our assumptions: Cognitive Empathy was positively correlated to all four justice sensitivities, strikingly also with victim sensitivity (Hypothesis 4), but not with pro-environmental behavior (*p* = 0.16; Hypothesis 6). Also, affective empathy was positively correlated with other-oriented justice sensitivities, but not with victim sensitivity (Hypothesis 5). The correlation between affective empathy and pro-environmental behavior was weak and positive, though this relation was not significant (Hypothesis 6; *p* = 0.05).

Pearson and Spearman values did not namely differ, except for two relations: Firstly, affective empathy showed a rather medium positive correlation with pro-environmental behavior, when calculated with Pearson (0.22, *p* < 0.001), but a non-significant weak correlation when calculated with Spearman (0.11, *p* = 0.05). Secondly, the small correlation of compassion and pro-environmental behavior was significant with Pearson (0.13, *p* < 0.005), but did not exist and was not significant with Spearman (0.09, *p* = 0.10). In line with our preregistration, we used Spearman for further interpretations as these variables were not normally distributed.

With a multiple regression analysis, it was possible to predict pro-environmental behavior (*F*(3, 323) = 7.26, adjusted *R*^2^ = 0.05, *p* < 0.001) with the three facets of empathy, but this did not support hypothesis 8, as compassion was not the strongest predictor (*β* = 0.15, *p* = 0.01). Cognitive empathy (*β* = −0.30, *p* < 0.001) and affective empathy (*β* = 0.29, *p* = 0.01) showed higher associations, though contrary to the direction we hypothesized.

As correlations of the three other-oriented justice sensitivities (perpetrator, beneficiary, and observer sensitivity) were in the same direction and similar size, we decided to create a variable called “sensitivity for others”. For this purpose, the mean of perpetrator, beneficiary, and observer sensitivity was calculated and used as an additional score. This is a common procedure in many articles dealing with justice sensitivity (e.g., Decety and Yoder, 2016). However, all exploratory moderation hypotheses (3, 9, and 10) had to be rejected, as no moderation effects were found. This is shown in Table 3.

## 4 Discussion

### 4.1 Interpretation and integration of results

The strong associations of perpetrator, beneficiary, and observer sensitivity with pro-environmental behavior are consistent with both our theoretical expectations and previous research (Köhler et al., 2024). In contrast to the mixed results reported by Nicolai et al. (2022), the use of the GEB scale—a validated and behaviorally predictive measure (Arnold et al., 2018; Kaiser, 2020)—likely enhanced the robustness of our findings. By comparison, victim sensitivity showed no significant, but negative association with pro-environmental behavior. Pro-environmental behavior, consequently, is linked to perceptions of injustice and moral responsibility. The decisive factor is who is perceived as morally relevant: who is responsible and who is disadvantaged (others vs. self).

Contrary to our initial expectation of a direct association, compassion was not significantly correlated with pro-environmental behavior at the bivariate level. However, when considered simultaneously with cognitive and affective empathy in a multiple regression model, compassion emerged as a significant positive predictor of pro-environmental behavior. This suggests that compassion may account for unique variance in pro-environmental behavior beyond other empathy facets, despite its weak zero-order association. Importantly, this effect was not replicated in the moderation model including other-oriented justice sensitivity and the interaction term. In this model, neither compassion nor the interaction between compassion and justice sensitivity significantly predicted pro-environmental behavior. Taken together, these findings indicate that the association between compassion and pro-environmental behavior is model-dependent and does not provide robust support for a direct or moderating role of compassion in the justice sensitivity–behavior link. One possible explanation for this pattern is that compassion shares variance with other empathy facets, such that its predictive value becomes visible only when overlapping components are statistically controlled. Alternatively, compassion may be more strongly related to specific forms of helping behavior than to the broader behavioral index assessed in this study.

Given that compassion is a well-established predictor of prosocial behavior, this pattern might also suggest that the motivational foundations of pro-environmental and prosocial behavior differ more strongly than previously assumed. Individuals may engage in high levels of pro-environmental behavior for fundamentally different reasons, such as concern for nature itself or, alternatively, concern for human wellbeing, without these motivations necessarily co-occurring. Pro-environmental behavior might not be universally construed as prosocial unless the social consequences of climate change are made salient. If individuals primarily perceive environmental action as nature-focused rather than human-focused, empathic concern toward others may not automatically be activated as a motivational driver. Thus, our results indicate that the moral framing of environmental issues may determine whether empathic concern becomes behaviorally relevant.

Regarding empathy, the negative association of cognitive empathy with pro-environmental behavior contradicts earlier findings (Ienna et al., 2022) and raises important questions. This challenges the notion that cognitive empathy is inherently linked to prosocial or moral behavior. It is possible that cognitive empathy as a skill may be applied in self-serving ways unless accompanied by affective engagement or normative orientation. In line with this, cognitive empathy, in this study, was present in both other- and self-oriented justice sensitivities. This implies, individuals high in victim sensitivity are also capable of perspective-taking—but they may do so primarily in service of their own perceived disadvantage. Maybe, here lies the real “dark side of empathy” and not, as in Breithaupt’s (2019) conception, in an over-identification with others. Roeser (2012) argues, that emotions play a crucial role in recognizing the moral significance of a situation. Affective empathy might thus be more decisive for translating perspective-taking into moral concern and, ultimately, into behavior aligned with collective goods like environmental protection than cognitive empathy.

However, the correlations of pro-environmental behavior with affective empathy were either small or absent, depending on the correlation coefficient applied. Importantly, our measure of affective empathy is conceptually closer to Davis´ IRI subscale empathic concern than to self-focused personal distress. A substantial body of literature demonstrates that empathic concern reliably predicts prosocial behavior by fostering other-oriented motivation and emotion-driven decision-making (Batson et al., 1983; Carrera et al., 2013; Decety and Cowell, 2014; Gaspar, 2016). From this perspective, our findings do not contradict existing research but rather suggest that the translation of empathic concern into pro-environmental behavior may depend on contextual factors. Future research should therefore examine more explicitly under which conditions empathic concern toward affected populations is activated in the environmental domain.

This aligns with previous research (Lockwood et al., 2014), which suggests that the positive effects of affective empathy on prosocial behavior are contingent on factors such as emotional regulation. In this regard, the integrative model proposed by Stevens and Taber (2021)—which combines cognitive empathy, affective empathy, and compassion with emotion regulation—offers a valuable framework for understanding the complex pathways linking empathy to behavior. According to their model, observing another’s distress elicits affective empathy through emotional transfer to the observer. At this stage, self-regulation becomes essential: when successful, it enables the expression of compassion and cognitive empathy, whereas failure results in personal distress and self-protective behavior. If compassion and cognitive empathy are activated, the individual evaluates the costs of helping. Excessive costs promote self-protective responses, whereas tolerable costs facilitate other-protective behavior, which may reduce the distress of others while providing relief or even self-reward for the observer. Stevens and Taber (2021) further point to potential interventions—such as emotion regulation training, compassion training, empathy efficacy training, and mindfulness training—that may strengthen self-regulation capacities. Yet, as the terminology and methods of these trainings vary considerably, further conceptual clarification is needed. While their model addresses prosocial behavior in general, future research should examine whether similar mechanisms extend to pro-environmental behavior.

Contrary to our hypotheses and previous findings (Decety and Yoder, 2016), affective empathy was positively associated with all three other-oriented forms of justice sensitivity. This may be due to their shared emotional responsiveness to others’ suffering: individuals high in affective empathy might perceive injustices more intensely and react more strongly to them. However, with this work we cannot conclude on the specific psychological process underlying here, which is why further research is needed here. A reason for the contrary findings of empathy facets and pro-environmental behavior in the article by Ienna et al. (2022) may be the operationalization of pro-environmental behavior: While Ienna et al. focused specifically on preservation behavior—typically characterized by the protection of natural areas or species—our study examined a broader range of ecological behaviors, including political engagement and sustainable consumption patterns such as a vegetarian diet. It is plausible that affective empathy, which involves emotional resonance with others, plays a more significant role in behaviors that are morally or socially charged, such as political activism or dietary choices, than in preservation behavior per se. This distinction suggests that the relevance of specific empathy facets may vary depending on the type of pro-environmental behavior under investigation. Future research would benefit from a more fine-grained analysis of behavioral subtypes to clarify the psychological mechanisms underlying each. However, impactful pro-environmental behaviors should be the focus of such investigations.

The lack of moderating effects of cognitive and affective empathy on the relationship between justice sensitivity and pro-environmental behavior might be explained by the consideration that justice sensitivity and empathy may function as independent constructs rather than amplifying each other. The emotional components inherent in justice sensitivity—such as moral indignation or guilt—may be sufficient to trigger pro-environmental action, reducing the incremental effect of empathy. Overall, our findings suggest that justice sensitivity and empathy may contribute to pro-environmental behavior in additive rather than interactive ways. However, this part of the analyses were explorative and null findings might also reflect limited power instead of an absence of effects.

Additionally, the measurement of empathy remains a methodological challenge. While we employed validated instruments to assess cognitive and affective empathy, the available tools may not fully capture the complexity of these constructs. Alternative operationalizations—such as behavioral tasks, peer assessments, or neuropsychological measures—might yield different results and provide a more nuanced understanding of the empathy–behavior relationship. Expanding the methodological toolkit in this domain could enhance the robustness and generalizability of future findings.

### 4.2 Insights for interventions on pro-environmental behavior

While empathy trainings have shown potential for increasing prosocial behavior (van Teding Berkhout and Malouff, 2016), their application to promote pro-environmental behavior remains largely unexplored. In light of the conflicting findings presented in this article relative to prior research, it is imperative to undertake further investigation into the conceptualization and measurement of empathy. Only if there is a clear and shared understanding of what exactly is meant by empathy, which psychological processes are involved, and how these processes influence pro-environmental behavior, does it make sense to develop elaborate, complex, and costly empathy training programs in this context. Both, compassion and affective empathy have shown to be strong predictors of prosocial behavior (FeldmanHall et al., 2015; Petrocchi et al., 2021). Therefore, this effect might depend on the specific context, such as framing pro-environmental behavior as prosocial behavior. A specific focus might be the explicit context in which these empathy facets modify behavior and whether this process is direct or indirect. However, this remains speculative as we can not conclude it directly from our data.

Our research suggests that interventions aimed at increasing pro-environmental behavior may benefit from targeting justice-related motives. However, it is important to note that our data reflect associations between dispositional tendencies rather than comparisons of interventions. As such, these assumptions warrant further investigation in future research. One promising direction could involve interventions that enhance other-oriented justice sensitivity or reduce victim sensitivity. For instance, Maltese et al. (2013) developed a training protocol designed to increase individuals’ tendency to interpret personal advantages as unjustified. This intervention led to greater willingness to sacrifice personal resources to restore justice, thereby increasing prosocial behavior (Maltese et al., 2013). A similar approach—framing climate change through a justice-based narrative—could potentially foster pro-environmental engagement. Encouraging individuals to reflect on their own environmental privileges, compare ecological footprints, and engage with narratives highlighting the unequal impacts of climate change might be concrete applications of such an approach and may let them come to reinterpret their advantages as unjustified. Framing pro-environmental actions as a means of restoring justice could thus foster stronger motivation to engage in pro-environmental behavior. Such justice-based interventions may be particularly effective when targeted at structurally privileged groups, such as university students in high-income countries, corporate or administrative decision-makers, educators, and youth from affluent backgrounds. These groups often remain distant from the consequences of climate change but possess significant potential for long-term impact through future leadership, institutional influence, and multiplier effects.

### 4.3 Insights for future research

Future research should further explore how justice narratives—especially in the context of global inequality and postcolonial structures—can activate other-oriented sensitivities. This may help design effective interventions for climate justice. Nevertheless, it is essential that a justice-oriented perspective also considers the accompanying emotional processes, since intense emotional arousal or cognitive dissonance may increase the risk of moral disengagement (Nicolai et al., 2022; Stoll-Kleemann et al., 2022). We argue that it is essential to provide ongoing support and guidance to individuals after exposing them to the realities of climate change, ensuring they are not left alone with potentially overwhelming emotions. Consistent with the integrative model proposed by Stevens and Taber (2021), societies must develop both, individual and collective coping strategies that enable the adaptive regulation of these emotions, thereby converting the affective responses elicited by justice sensitivity into impactful pro-environmental behavior. One initial approach in this direction are so-called climate cafés—appointments, which offer a structured space for individuals to share and process difficult thoughts and emotions related to climate change.

Our findings also point to the relevance of examining affective processes in relation to justice sensitivity. Although cognitive empathy did not show the expected correlations, both affective empathy and compassion correlated positively with other-oriented justice sensitivities. This raises the possibility that it is not perspective-taking per se, but rather emotional resonance with others’ disadvantage, that motivates moral concern and environmentally responsible behavior. In this sense, emotional awareness may function as a moral amplifier—triggering shifts in perspective toward collective justice.

In this article, we examined dispositional justice sensitivity at the individual level. However, justice perceptions may also be shaped by social identities and, consequently, by ingroup–outgroup mechanisms. Future research could incorporate this perspective, as we did not identify any empirical studies addressing ingroup–outgroup dynamics in justice sensitivity. However, an international comparison has shown that individuals in collectivist countries showed higher beneficiary sensitivity, rendering individuals more sensitive to their own advantages in the context of others’ suffering (Wu et al., 2014). Investigating the interplay between micro- and meso-level processes of justice sensitivity is particularly important in light of global crises characterized by substantial spatial injustice. With regard to the question whether pro-environmental and pro-social behaviors can be conceptualized as two branches of the same underlying system, we got contradictory findings. On the one hand, compassion is highly associated with prosocial behavior in literature, but not with pro-environmental behavior in this study. On the other hand, the relevance of other-oriented justice sensitivity in both, pro-environmental and prosocial behavior, suggests that they may be grounded in distinct yet overlapping motivational structures. Future research, potentially using qualitative approaches, could provide deeper insight into the diversity of motivations underlying pro-environmental behavior and show, for example, whether pro-environmental behavior is conceptualized as prosocial.

### 4.4 Limitations

Our sample consisted predominantly of German-speaking university students, most likely resulting in a WEIRD sample (Western, Educated, Industrialized, Rich, Democratic). This limits the generalizability of our findings, especially regarding the role of justice sensitivity. In non-Western contexts, the interpretation and behavioral consequences of justice-related constructs may differ significantly. Victim sensitivity, for instance, may not necessarily correspond with real-life disadvantage but might reflect perceived threats to privilege or status. In our sample, high victim sensitivity could partially reflect what DiAngelo (2022) terms “white fragility”—a defensive reaction to challenges to unacknowledged social advantage.

Furthermore, while we used validated self-report instruments to assess empathy, justice sensitivity, and pro-environmental behavior, future research should incorporate alternative methods. Empathy could be measured through third-party evaluations or behavioral tasks, while pro-environmental behavior could be assessed using observation or ecological momentary assessment to capture real-life behavior. Moreover, employing more specific constructs, such as personal distress and empathic concern, may be theoretically more informative than relying on the broader labels of cognitive and affective empathy. Additionally, longitudinal designs or experimental interventions would help to clarify causal mechanisms.

Lastly, this study relied on a convenience sample consisting primarily of university students. While this limits the generalizability of the findings to broader populations, it is worth noting that university students represent one of the two target groups in which empathy trainings have been shown to be particularly effective (van Teding Berkhout and Malouff, 2016). Therefore, although this sampling method poses a methodological limitation, it may be less problematic in the specific context of this research, which explores the potential of empathy-based interventions to foster pro-environmental behavior.

### 4.5 Conclusion

While previous research has often emphasized empathy as a driver of prosocial and pro-environmental behavior, our findings suggest that this relationship is more nuanced and depends on the moral frameworks through which environmental issues are interpreted. The present results highlight the importance of differentiating between empathy facets and examining the conditions under which they translate into environmental engagement. As Gaspar (2016) argues, empathic concern alone does not automatically translate into universal moral action; rather, its motivational force may remain selective unless it is embedded within broader normative structures—such as justice-related beliefs—that extend concern beyond immediate interpersonal contexts. Consistent with this perspective, other-oriented justice sensitivities showed strong and consistent associations with pro-environmental behavior, underscoring the pivotal role of justice-based appraisals in motivating environmental engagement.

Based on these results, we draw two main conclusions:

(1) The current evidence base is insufficient to support broad claims about the pro-environmental relation with various facets of empathy. While empathy is often assumed to enhance environmental concern, our cross-sectional study findings suggest that its associations are nuanced and likely dependent on the specific context in which empathy is activated. To better understand these mechanisms—and to uncover potential unintended side effects—future research should adopt a more differentiated perspective and employ diverse methodological approaches.
(2) In contrast, justice sensitivity—particularly in its other-oriented forms—consistently shows empirical associations with pro-environmental behavior. Based on its robust theoretical foundation, future research might find effective intervention strategies using this relation. For example, making justice concerns more salient in climate communication, especially by highlighting who benefits from the current injustices, may be useful here. However, such framing also evokes strong emotional reactions, such as anger, guilt, or helplessness. These emotions must not be overlooked: society must provide spaces and strategies for individuals to process and regulate these feelings in a constructive way. If people are left alone with emotionally charged responses to climate injustice—triggered by facts, images, or narratives—this can lead to overwhelm and ultimately to moral disengagement. Acknowledging and supporting emotional coping is therefore a critical element of justice-based climate communication.

## Data Availability

The datasets presented in this study can be found in online repositories. The names of the repository/repositories and accession number(s) can be found in the article/supplementary material.

## Footnotes

1 This reliability index is calculated within the framework of Rasch modeling and is conceptually similar to Cronbach’s alpha (see Kaiser, 2020).

2 Not part of this article.
